# Navigating truth and disinformation: A comparative analysis of generational responses to the 6 February 2023 earthquake in digital media in Türkiye

**DOI:** 10.1016/j.heliyon.2024.e38667

**Published:** 2024-09-27

**Authors:** Yasemin Bilişli, Fatma Çakmak, Selin Aygen Zetter, Mehmet Ilgaz Ünal

**Affiliations:** a Department of Office Services and Secretariat, Social Sciences Vocational School, Akdeniz University, Antalya, Türkiye; b Fethiye Faculty of Business Administration, Muğla Sıtkı Koçman University, Muğla, Türkiye

**Keywords:** Crisis communication, Digital literacy, Disinformation, Fake news, Intergenerational differences, Qualitative research, Thematic analysis, Türkiye's February 6, 2023 earthquake

## Abstract

This study aims to examine how different generations perceived and responded to news and disinformation about the February 6, 2023 Earthquake in Türkiye, focusing on their trust in news sources and methods of verifying authenticity. In this study, the data were collected from semi-structured interviews held with 30 participants using qualitative methods and they were analyzed with MAXQDA Analytics Pro 2022, through thematic analysis to uncover generational nuances in digital media engagement and trust. The analysis revealed five primary themes: digital media usage habits, trust and reliability in news sources, fake news verification practices, causes of fake news, and views on media legislation. The findings of the study indicated significant generational differences in digital media consumption habits. Notably, maintaining consistent online presence and integrating digital media into everyday life in Generation Z stood out as decisive factors in their reactions to news and disinformation about the February 6, 2023 Earthquake. The study also highlighted varied approaches among generations toward detecting disinformation. While Generation X preferred to use the methods of verification over broadcast media, Generations Y and Z showed a propensity for utilizing digital tools for identifying and verifying fake news. Attitudes toward media legislation differed among generations, yet there was a general consensus on the necessity of such laws to adapt to the digital age's challenges and play a crucial role in combating disinformation. This study offered a detailed comparative analysis on how different generations use digital media and their attitudes toward accuracy of news, particularly in response to significant events such as the February 6, 2023 Earthquake in Türkiye. This study would contribute to adopt a deeper understanding about the critical role of accurate information access during crises and the varying media consumption habits and attitudes toward disinformation across generations. The study emphasized the importance of tailored approaches in media literacy education and disinformation counter-strategies, as well as the need for media laws to be updated in accordance with the demands of the digital era.

## Highlights

• Investigates generational responses to the 2023 Turkey earthquake news and disinformation across Generations X, Y, and Z.
• Utilizes qualitative interviews with 30 participants to explore media consumption and trust in news sources.
• Highlights Generation Z's digital presence and contrast with Generation X's traditional news verification.
• Reveals consensus on updating media laws to combat misinformation, emphasizing the need for legal frameworks.

## 1 Introduction

The rapid spread of digital media in the 21st century has transformed how information is shared and consumed, blurring the lines between truth and falsehood and complicating efforts to assess content accuracy [1]. Despite the extensive usage of digital media, there is a limited number of studies on how different generations including well-educated Turkish people perceive and use digital media for accurate news consumption. This study addresses this gap by examining how well-educated Turks from Generations X, Y, and Z accessed and verified news during the Türkiye's February 6, 2023 Earthquake, which resulted in 50.000 deaths. Understanding intergenerational differences is crucial for developing effective crisis communication strategies tailored to different age groups, thereby enhancing public response during natural disasters.

Three main research questions were determined to seek answers.

Main Research Question 1 (RQ1) : How do different generations (X, Y, and Z) utilize digital media for news consumption?

Sub-Question (RQ2) : How do these generations react to the earthquake-related news received through digital media?

Main Research Question 2 (RQ3) : What levels of disinformation awareness exist among generations X, Y, and Z concerning earthquake news?

Sub-Question (RQ4) : How do these generations verify the accuracy of earthquake-related news they encounter?

Main Research Question 3 (RQ5) : What are the opinions of Generation X, Y, and Z on the disinformation law?

According to Shanmugasundaram and Tamilarasu, the fact that different age groups use digital media with different frequencies and skill levels has a significant impact on content production and consumption [2]. Houston et al. [3], state that during crises, the need for accurate information and efficient communication increases, thus spotlighting the daunting challenges in securing both accuracy and reliability within digital media platforms [3]. In particular, the two consecutive earthquakes occurring in Türkiye in February 6, 2023, 9 h apart, and the disinformation encountered on digital news platforms related to these events revealed how critical access to accurate information is.

Understanding how different generations responded to disinformation during the February 6, 2023 Earthquake in Türkiye is crucial due to several reasons. Intergenerational differences in media consumption habits, shaped by technological advancements, influence how disinformation spreads and is perceived across platforms. For example, Generation Z, as digital natives, heavily relies on social media for news; whereas, Generation X may still prefer to use broadcast media sources. Analysis of these differences can enable to identify which sources are deemed more credible by each generation and how this affects their response to crisis-related information. This understanding is essential for developing targeted strategies to combat disinformation.

During crises like earthquakes, the reception and processing of information can significantly influence public behaviors. By studying generational responses, we can tailor communication strategies to ensure access to accurate information and that information is trusted by all age groups, ultimately enhancing public safety and response efforts. Insights obtained from this study can inform policymakers and educators about the need for tailored media literacy programs. Understanding intergenerational differences allows to develop targeted interventions in order to improve digital literacy and resilience against disinformation.

This study specifically analyzes public reactions to digital media's portrayal of earthquake news and examines the awareness of disinformation among Generations X, Y, and Z. By holding in-depth interviews with 30 participants from these generational cohorts, the study seeks to uncover their perceptions of earthquake news, their evaluative processes, and their methods of verifying news accuracy. The aim of the present study is to gain an elaborated understanding of how different generations navigate information during crises through this qualitative approach by offering valuable insights for enhancing media literacy and information resilience in the digital age.

Following steps were followed in organization of the present study. First, we defined our key terms—digital media usage habits, fake news, and disinformation—by providing historical and contemporary context for each. Then, we outlined our framework for examining intergenerational differences in digital media engagement and trust, followed by a description of methodological approach used in the present study. Afterwards, we then presented our findings through five main themes: Theme 1, Digital Media Usage Habits; Theme 2, Trust and Reliability in News Sources; Theme 3, Fake News Verification Practices; Theme 4, Causes of Fake News; and Theme 5, Views on Media Legislation. Each theme was supported by detailed analysis and statements of the participants. Finally, we discussed the implications of our findings for understanding intergenerational differences in media consumption and developing effective strategies to combat disinformation and enhance media literacy in digital environments.

## 2 Background

### 2.1 Conceptual framework of disinformation

The current era is described with terms of digital age, information age, communication age in relation to technological transformations. However, the proliferation of information happening owing to these technologies has also led to significant information pollution. The false and potentially harmful dimensions of media content result in the classification of information pollution into three types: Misinformation, malinformation, and disinformation. Misinformation is the unintentional, unwitting sharing of incorrect or incomplete information, but it is not intentionally produced and is not intended to cause harm. Malinformation is the sharing of true, accurate information with the intention of causing harm. It usually takes the form of leaking accurate information in order to benefit a person, organization, or country, or to cause some to lose power. Disinformation, on the other hand, involves the deliberate creation and sharing of false information with the intent to harm a person, social group, organization, or country [4]. Disinformation refers to the most complex and perhaps the most maliciously shared content among these concepts. It consists of deliberately disseminated information distortions and is produced by creating suspicious and false controversies for economic gain or ideological advantage [5].

In the World Economic Forum (WEF)'s Global Risks Report in 2018; online disinformation is described as one of the most important global risks to environmental, economic, technological, and institutional systems [6]. One of the risks associated with disinformation is associated with arbitrary framing of content. For example, the government suggests any content it does not like as disinformation. As a matter of fact, in Ref. [7]Hayward's article “The Problem of Disinformation: A Critical Approach” published in 2024; it is argued that disinformation can certainly be used instrumentally to discredit views that the speaker disapproves of.

In her work titled “Unnatural Ecologies: Environmental Metaphor in Media Theory”, Heise, (2002) states that her “media ecology” metaphor regarding information pollution is quite striking. Claiming that we live in the sea of information, she expressed this situation with the metaphor of “media ecology”. This analogy operates with two layers of metaphorical meaning. On the one hand, metropolises and their built environment created upon the development of technology are conceived as an ecosystem; on the other hand, this ecosystem itself forms part of a larger ecological network of information exchange. At this point, media is not only the tools that people use, but also a structure in which people act with their perceptions, forms of discourse, and social behaviors along with the emergence of communication technologies [8].

Wardle and Derakhshan [4] state that three primary factors contribute to the emergence of information pollution. Firstly, the agents or intermediaries who create, produce, and/or distribute information come into play. The identity of these agents or intermediaries and their motivations for creating content are essential in the formation of information pollution. Secondly, the nature of the message itself is crucial. The message's type, format, characteristics, and structure and what is emphasized or relegated to the background are all important parameters. Lastly, the interpreter, namely the recipient of the message, plays a significant role in information pollution. How the recipient interprets and consumes the content, and accordingly acts is another key factor in the emergence of information pollution [4].

When it comes to intermediaries that create, produce or distribute information; in the study titled “Who is monitoring the World Health Organization? “Post-truth” moments beyond infodemic research” by Noakes, Bell and Noakes [9] about the infodemic they associated with the COVID-19 pandemic in 2022; they pointed out that academics who produced knowledge (related to the subject in their articles) remained under the influence of political leaders and governments more than epidemiologists during the pandemic crisis. In the situation called as post-truth taking this explanation further, they argued that whistleblower policies including fact-checking, appealing to emotions, and denying science resulted in the return of fascism. Similarly, in their study, Coman et al. [10], argued that during crises, mass media served as a bridge between political actors and people and ultimately joined governments in shaping the narrative of the crisis [10].

Governments and oppositions have continued their propaganda and political communication in parallel with the information technologies of the age in power struggles throughout history and all over the world. Especially in times of crisis, these forms of communication are at more critical levels and can escalate the crisis. Similarly, in the Türkiye's February 6, 2023 Earthquake, the government and the opposition had different approaches toward this crisis. As a matter of fact, considering that media outlets also report on pro- or opposing policies, it is can be asserted that they produce and share contents under the influence of their ideological views. Therefore, as expressed in the study by Noakes, Bell and Noakes; it can be said that news about the Turkish earthquake crisis was affected by politicians, just like the COVID-19 pandemic.

According to Thomson et al. [11], disinformative content is more common in polarized countries where inequalities and disenfranchisement are prevalent. It serves to distract, confuse, manipulate, and divide people and cause dissonance and uncertainty. More false and attracting content is given instead of real but uninteresting content by taking advantage of people's turn to sensational events due to economic reasons. Moreover, the speed at which content is published and shared daily, the visual literacy of those consuming this content, economic realities, and ideological differences are effective in the spread of disinformation in mass media and especially in digital mass media [12].

Although some explanations and information about disinformation are clear in the literature, Dutton [13] states that internet media is the fifth power. In addition to giving power to some individuals and groups, the complex structure of the internet environment also includes certain conditions such as enabling the sharing of contents against the public and creating prohibitive and restrictive rules and laws [13]. This has all brought about uncertainty concerning what content is or is not considered disinformation. As a matter of fact, similar discussions have come to the fore in recent years in Türkiye regarding the disinformation law. Particularly, opposition parties and opposition media outlets have emphasized this uncertainty, and it has been claimed that the government will punish and prevent opposing views under the name of the disinformation law.

Adopting the “Fifth Estate” concept of digital media, Dutton [13] asks how behaviors and actions are organized online to challenge and question those in power. In doing so, he argues that the “Fifth Estate” is not defined by the use of the internet per se, but the strategic use of the internet by individuals to increase their communicative power. While emphasizing the interest of the public-spirited networked citizens of the digital age in using the internet for the public good, he argues that the Fifth Estate is a largely progressive force with the potential to “shape accountability and governance in politics and society” and a space where individuals and groups can directly challenge their political representatives [13].

An important feature identified by Dutton is that the Fifth Estate can bring a new set of actors into the political process. These actors bring marginalized and overlooked individuals and groups to the fore. They are also a source of threat to the Fifth Estate. In this sense, the increase in socially harmful content provides the basis for regulatory efforts and policies to impose the control of a large part of internet traffic, such as the shutdown of the internet or the use of access restrictions to sites. Given the efforts of state and private actors to create certainty and exert control, such spaces can be seen as threatening, just as they were when the Fourth Estate emerged.

### 2.2 Digital natives, digital immigrants, digital hybrids, and digital literacy

Çakır states that generations have emerged throughout the history of mankind. Generation refers to people who are born in almost the same time period and live in a certain economic, social and social structure. Since societies are subjected to the constant change and transformation, nothing remains constant. These changes and transformations differ the characteristics, functions, roles, and life perceptions of generations. Generations reflect the changing world and societies [14]. [16].

Technological developments have transformed social structure and communication styles, causing radical changes in individuals’ lifestyles and personality traits. Generations X, Y, and Z from different historical contexts and experiences exhibit different characteristics in their interactions with digital media.

Generation X, born between the mid-1960s and early 1980s, have experienced significant political and technological changes. In Türkiye, this generation grew up during the political turmoil induced by the 1980 military coup and then the economic reforms of the 1980s and 1990s, which have made Generation X more cautious and selective in their media consumption habits [15]. The related studies have revealed that this generation prefers to use broadcast media but can also adopt digital platforms when necessary [16].

Millennials or Generation Y, born between the early 1980s and mid-1990s, grew up during the rise of the internet and social media. In Türkiye, they witnessed the digital revolution, economic liberalization, and significant political changes, including the 2001 financial crisis and the rise of social media in the early 2000s. This generation values immediacy and accessibility in digital platforms, balancing their trust between digital and broadcast media sources [17]. They are characterized as digital hybrids, adept at navigating both digital and broadcast media environments [16].

Generation Z, born between the mid-1990s and early 2010s, are digital natives. This generation has integrated digital media seamlessly into their daily lives, exhibiting a preference for social media platforms and digital tools for information verification. Growing up in Türkiye, Generation Z has been influenced by the rapid spread of smartphones and social media, making them highly proficient in using these technologies for news and social interaction [15].

Digital natives refer to the new generation of students who are born into a technology-based world and develop a technological learning language from a very young age [18]. Digital natives are connected with the virtual environment from the moment they are born and this connection is considered natural. While the concept of “Digital Native” proposed by Ref. [19] has been widely influential, it has also been considerably criticized. In their study entitled “Debunking the ‘Digital Native’: Beyond Digital Apartheid, Toward Digital Democracy”, Brown and Czerniewicz [20] argue that the concept of digital natives does not account for significant inequalities in digital access and literacy among different socio-economic groups [20]. They argue that this concept can create a kind of “digital apartheid” where only elites have full access to digital media and its benefits. Palfrey and Gasser [21] state that the digital tools connecting digital natives are their primary means of organizing human relations. Such use of technology creates a new form of knowledge, but most importantly, “the 24/7 network they have created by combining technology and human, in a way that has never happened before, has fundamentally transformed human relationships” [21].

All of these also indicate that digital natives have developed digital literacy. In this case, they are expected to be aware of disinformation. According to Tutiasri & Febriyanti, generation Z, called as digital natives, has a better media literacy compared to the other generations [22].

Generational differences also affect news credibility [23]. Metzger and Flanagin's [24] study, reported that internet experience or skill may also influence the amount of effort people are willing to expend on online credibility assessment. Digitally literate individuals are more likely to use verification methods because they can verify information from multiple sources [24].

In the study conducted by Escoda et al., in Spain, they found that Generation Z's media and information consumption, their use of social networks and their relationship with fake news were all related to trust/confidence. Their results showed that young Spaniards used networks to get information and lacked trust in social networks, they consumed the most [25].

Digital immigrants are in their twenties and later and are acquainted with technology, the internet, and the web. This concept describes individuals with a lower level of technology literacy compared to digital natives, who may encounter difficulties or various adaptation problems in the use of technological tools and technology-based learning [19]. “Digital immigrants – in contrast to digital natives – are not significantly born into and live in a digital world, but they find their way in the digital world” [21]. They primarily prefer printed materials to obtain information. They tend to resort to a guide or manual in using any technological product or program. While digital natives prefer web-based research methods, digital immigrants prefer to go to libraries [18]. Digital immigrants use digital media for specific purposes and needs [26]. Therefore, it can be asserted that digital immigrants have low levels of digital literacy.

Digital hybridity was put forward by Palfrey and Gasser based on the fact that the habits of individuals in society to use digital technologies should be determined by not only age but also cultural factors. According to study by Tkhostov et al. [23]; generation Z favors a fact-checking strategy to identify news reliability, while Generation Y relies on offline contacts. In this case, digital hybrids are expressed as those who try to adapt themselves to the newly formed environment, are preparing for the change process, are changing, and continue their old habits while changing [27]. Thus, it can be stated that the digital literacy of digital hybrids is above a certain level.

In the current era where all kinds of information pollution exist, it has become almost a necessity for the masses to acquire the necessary literacy along with the advancing technology. In this sense, the concept of digital literacy has emerged. Although there are many definitions of digital literacy in the literature, Gilster and Gilster [28] define digital literacy as one's knowledge and ability to access and use various hardware devices and software applications, competence to understand and critically analyze digital content and applications, and the ability to create digital content with digital technology.

### 2.3 “The disaster of the century” in Türkiye and crisis communication

In Türkiye, an earthquake with a magnitude of 7.7 occurred in Pazarcık district of Kahramanmaraş province at 04.17 on February 06, 2023 and affected 10 provinces including Hatay, Adıyaman, Malatya, Adana, Gaziantep, Şanlıurfa, Diyarbakır, Osmaniye, and Kilis. At 13.24, 9 h after this earthquake, another 7.5 magnitude earthquake hit the Ekinözü district of Kahramanmaraş province. As a result of both major earthquakes, approximately 50 thousand people lost their lives and more than 129 thousand people were injured. These earthquakes have been described as the “disaster of the century”.

many parts of the world, especially Türkiye expressed great sadness about the disaster that caused such destruction. Many countries sent their aid teams. The magnitude of the disaster and the resultant devastation were undoubtedly on the agenda in the world press for days. However, this era has reached such a point that mass media-wise technology, news production and sharing has not been limited to news professionals and online content creation has been started by citizens living in and going to the region.

There are 74.41 million internet users and 57.50 million active social media users in Türkiye, accounting for 86.5 % and 86.8 % of the population over the age of 18, respectively [29]. This rate signifies that a very large proportion of the population is exposed to digital content in case of a crisis such as a disaster. Several factors such as widespread use of digital mass media, magnitude of the earthquake, a large area affected by the earthquake, and the disruptions in crisis management further increased the chaos in the region, as is reflected in news content.

Generally, information pollution occurs more in times of crisis [30]. As more and more contents are shared in the desperation of suffering, disinformation is deliberately generated to gain ideological and personal benefit from the chaos. The viral effect of this situation leads to new crises. At this point, this study was conducted with the need to determine how the masses receive the news content and approach to disinformation.

Correct crisis management and crisis communication are of great importance in times of crisis that arise suddenly and must be resolved urgently; otherwise, causing great material and moral damage. Media plays a major role in establishing communication. Meyer [31] points out that target audiences are informed through the media and announcements are made to the public and stakeholders about innovations. Teufel et al. [32], state that in times of crisis, politicians send messages not only about their own positions but also inform the public and call for action to prevent the crisis [32]. In their study on media attention to earthquake risks, Opperhuizen et al. [33], showed that regarding certain risks, media served as strategic tools for networking. They suggest that in times of crisis, particularly high-profile political commentators can wield extra influence [33].

Some studies in the literature addressing crisis communication in disasters in the context of digitalization have mentioned about the benefits of social media in disaster management [[34], [35], [36], [37], [38]] In addition, Lestari et al. [39], indicate in their study that social media was the main source of information for crisis communication in disasters, with a significant percentage of the population relying heavily on social media platforms to receive information during disasters [39].

In the study conducted by Ata [40] on the February 6, 2023 Earthquake in Türkiye, He stated that the “information and social memory” features of the posts about the earthquake came to the fore. In the study by Unal & Sezer [38] on the same earthquake, they concluded that people considered social media as a communication and problem-solving tool.

On the other hand, Aydın [41] claimed that teyit.org, a verification platform operating in Türkiye during the February 6, 2023 Kahramanmaraş Earthquake, identified fake contents addressing claims that frequently appeared on social media. One of the shared contents revealed that a post from a page pretending to be the social media account of Turkish Influencer Oğuzhan Uğur, the owner and host of the internet channel Babala TV, which read “there is no state …, there is only Haluk Levent's Ahbap” was a montage. Another news article reported that the image of Kerem Kınık, the president of the Red Crescent, standing in front of a tent with “all credit cards are valid” written on it was an example of disinformation since it was created by combining 2 separate old images [41].

Similarly, Coşkun [42] showed in his study that many false and fabricated news were circulated on social media since the first day of the February 6, 2023 earthquake. One of these news was the post made by the user @seribere on X (formerly Twitter) on his social media account as “We have passed the conspiracy theory, here is the moment when Hatay was hit by titanium arrows, we thought it was an earthquake, we are under attack”. In the post, it was claimed that the black line crossing the sky was the titanium arrow that triggered the earthquake and further investigation proved that the black line was electric wires [42].

In the study by Argın [43] on the same earthquake, it was pointed out that X (formerly known as Twitter) was important in conveying messages such as calls for solidarity and cooperation, information about humans and animals under the rubble, sharing news, information and announcements, and ensuring coordination, but it also contained criticism of many institutions, ethical violations, and false content [43].

A study by Aşan [44] on the Kahramanmaraş earthquake reported that the reliability of social media accounts during a critical process such as disaster was a critical area of concern, especially in the context of disinformation, credibility assessment, and the spread of low-trust content.

Social media algorithms play an important role in determining what content will be popular and viewed widely. These algorithms are designed to maximize user engagement by promoting contents that evoke strong emotional responses, regardless of being positive or negative. A related study indicated that controversial and emotional content was more likely to influence users, leading to longer time spent on the platform and more interactions such as likes, shares, and comments [45].

According to Gillespie, [46] content creators, including influencers, are aware of these algorithmic preferences and often adapt their contents to use these mechanisms. By producing controversial or highly emotional content, they can increase their visibility and reach a wider audience. This practice, known as algorithmic manipulation, is a strategic way to get more clicks and engagement, ultimately leading to higher revenue and impact [46].

Serin and Ünlü [47] drew attention to two important disinformation news in their study. These were the false news that “Hatay Yarseli Dam exploded” on February 10, 2023 and “volcano erupted in Kahramanmaraş Kuşkayası” on February 11, 2023. These news reports, which were also announced by Turkish influencers, caused great panic and resulted in loss of time, people and equipment. Hundreds of thousands of people tweeted to the authorities asking for help, but after the news turned out to be disinformation, it was claimed that the news was spread for theft and looting. Even as disinformation was being fought, new false reports emerged [47].

Disinformation content circulated on social media is used to ensure that it is shared more by using these algorithms. Thus, it reaches many people in a very short time with its viral effect by support of the number of clicks, shares, likes and comments of disinformation.

## 3 Materials and methods

This qualitative study focuses on the February 6, 2023 Earthquake in Türkiye as well as the perception of this event through the media. The choice of qualitative methods over quantitative approaches was deliberate since it was aimed to capture the depth of personal experiences and subjective views across different generations concerning their reactions to earthquake news. In the study by Creswell [48], it was reported that qualitative research facilitated the collection of in-depth data, allowing for a profound understanding of complex human behaviors and attitudes [48]. This method enables to examine intergenerational differences by improving authentic voices, experiences, and perspectives and offering nuanced insights into how each generation interacts with media and processes information about such critical events. This study aimed to examine how Generation X, Y, and Z across Türkiye reach toward earthquake news, whether they accept or reject such news, and how sensitive they are to disinformation on digital news platforms. The data was obtained by holding semi-structured in-depth interviews (IDIs) with 30 participants. After the IDIs were transcribed, they were analyzed using MAXQDA Analytics Pro 2022 software. Thematic analysis was conducted in stages, starting with initial code generation from raw data, followed by theme development to capture underlying patterns. This approach ensured both inductive insights from the data and alignment with existing theoretical frameworks. Through inductive coding, thematic analysis enabled to identify gaps between generational perception and understanding about earthquake news and fake news [49]. The interview guide was designed by the research team specifically for the study. This guide includes a list of open-ended questions about attitudes toward earthquake news, strategies to counter fake news, and the use of digital media platforms. It was pilot-tested on a few participants before the study. The Supplementary File includes the full list of questions (table S1).

### 3.1 Participants and Procedure

#### 3.1.1 Sampling

The IDIs aimed to investigate the Generations X, Y, and Z's responses to disinformation within the context of their engagement with digital news platforms. To ensure a comprehensive understanding of the responses, the participants were selected based on various socio-demographic characteristics such as age group (representing Generations X, Y, and Z), educational level, place of residence, and occupational status in order to create a heterogeneous group that could potentially exhibit varying degrees of sensitivity to disinformation in the context of their interaction with digital news platforms regarding the earthquake. The interviews were conducted once to maintain the authenticity of participants' initial reactions and perspectives.

The sample of the study consisted of university-educated individuals, deliberately chosen to cover three distinct generations. The participants with higher educational level are presumed to offer more nuanced insights into their digital media consumption patterns due to their educational backgrounds. These participants are likely to provide more informed and detailed feedback on their use of digital media and sources of news. Furthermore, this group often has access to a broader variety of media outlets, which allows them to offer a wider perspective on their news consumption habits across different media platforms.

In this study, criterion sampling, a non-probability sampling method, was used, taking into account the limited population reach. In criterion sampling, the most appropriate participants for the research purpose are selected. The process was initiated through the references provided by the selected units through snowball sampling method. The sample consisted of a total of 30 individuals including 10 from each of Generations X, Y, and Z. In this context, the inclusion criteria were determined as follows: Actively following digital media, being over the age of 18, belonging to certain generations (for Generation X born between 1965 and 1979, Generation Y born between 1980 and 1999 and Generation Z born after 2000) [50], and voluntarily participating in the study.

The age range of participants in Generation X varied from 44 to 59 years, with a mean age of 51.4 years. The age range of those in Generation Y varied from 24 to 43 years, with a mean age of 36.6 years. Those in Generation Z were aged between 19 and 23 years, with a mean age of 21.8 years. Each generation group included a total of ten individuals as five females and five males. The educational backgrounds of the participants were consistent across age and gender groups. Their expressions were meticulously analyzed to reflect their socio-demographic categories accurately. This diverse representation aimed to ensure a balanced view across generations in terms of interaction with digital news concerning disinformation. Table 1 shows the socio-demographic characteristics of the participants.

**Table 1:** Socio-demographic characteristics of the participants.

Participant	Biological Sex	Age	Educational Status	Employment Status
**X-1**	Male	54	Bachelor's degree	Health officers
**X-2**	Male	53	Bachelor's degree	Doctor
**X-3**	Male	52	Ph.D. degree	Senior manager
**X-4**	Female	49	Master's degree	Engineer
**X-5**	Female	44	Bachelor's degree	Doctor
**X-6**	Female	52	Master's degree	Senior manager
**X-7**	Female	49	Bachelor's degree	Housewife
**X-8**	Female	53	Associate degree	Beautician
**X-9**	Male	49	Bachelor's degree	Tourism professional
**X-10**	Male	59	Bachelor's degree	Tourism agent
**Y-1**	Male	35	Master's degree	Academician
**Y-2**	Female	33	Bachelor's degree	Civil servant
**Y-3**	Male	42	Associate degree	Civil servant
**Y-4**	Male	34	Associate degree	Security guard
**Y-5**	Male	24	Associate degree	Tradesman
**Y-6**	Female	37	Bachelor's degree	Sales consultant
**Y-7**	Female	37	Bachelor's degree	Banker
**Y-8**	Female	43	Bachelor's degree	Banker
**Y-9**	Female	39	Bachelor's degree	Tourism professional
**Y-10**	Male	42	Bachelor's degree	Tradesman
**Z-1**	Female	23	Associate degree	Student
**Z-2**	Female	19	Associate degree	Student
**Z-3**	Female	20	Bachelor's degree	Student
**Z-4**	Male	23	Bachelor's degree	Student
**Z-5**	Male	21	Bachelor's degree	Student
**Z-6**	Male	23	Bachelor's degree	Student
**Z-7**	Male	21	Associate degree	Student
**Z-8**	Female	22	Associate degree	Student
**Z-9**	Male	23	Bachelor's degree	Student
**Z-10**	Female	23	Associate degree	Student

During the recruitment phase, five individuals either declined to participate or withdrew from the study after initially agreeing, due to time constraints, privacy concerns, or a lack of interest in the study's subject matter. It was observed that data saturation was achieved, indicating that additional interviews did not yield new themes or insights related to the study's objectives.

#### 3.1.2 Setting

The interview process was conducted from April 15, 2023 to May 15, 2023. The majority of the interviews were carried out face-to-face, either in the participants' homes or in locations they deemed safe and comfortable, such as cafeterias. Non-participants were not present during these interviews. This ensured that the discussions were conducted exclusively between the participants and the researchers. Such a setup facilitated a more personal and engaging dialogue and allowed the researchers to explore the participants’ perceptions and experiences more in depth as well as helped to maintain privacy and encourage open communication.

Each interview, lasting for approximately 30 min, was designed to efficiently cover key topics while ensuring depth and breadth in responses. To ensure confidentiality, the participants were anonymized and numbered by their generational category (e.g., X1). The semi-structured nature of the interviews allowed for a natural and fluid conversation flow. All interviews were recorded and meticulously transcribed for detailed analysis, preserving the original speech patterns, including grammatical errors and repetitions. However, statements of the participants were translated into English and grammatically corrected, and redundant phrases were removed to facilitate readability of the article. Following the interviews, transcripts were shared with the participants to solicit their feedback by ensuring the accuracy and integrity of their responses.

#### 3.1.3 Quality criteria

This study was conducted in accordance with the Consolidated Criteria for Reporting Qualitative Studies (COREQ) framework [51]. In the data analysis, the media usage habits of the participants were assessed and then it was scrutinized how they engaged with news content, exploring its relation to the generational interpretations.

The study was conceptualized and overseen by Y.B. The interviews were executed by S.A.Z., M.I.Ü., Y.B., and F.Ç., The interviewers conducting the research were composed of three academicians specialized in communication and one manager, all of whom received university education in qualitative research methods and have a deep knowledge and experience in digital media, news accuracy, and fake news topics. The researchers have adopted a reflective approach regarding their personal interests and potential biases toward the subject matter, aiming to maximize the objectivity and reliability of the study. Y.B. and F.Ç., conducted the coding and thematic analysis by ensuring a robust and methodical examination of the data. This study strictly adhered to the COREQ framework for qualitative research by paying attention to participant dynamics and data integrity. Details on the adherence to each of the 32 COREQ items are elaborated in the Supplementary File (table S2), sh owing our commitment to methodological rigor and transparency.

### 3.2 Data analysis

#### 3.2.1 Codes and themes

The coding process was conducted inductively, drawing directly from the research material. The obtained coding tree covered various critical domains, including patterns of digital media usage, trust in news sources, encounters with and identification of fake news, methods employed for news verification, and the social and political causes and impacts of fake news, as well as perspectives on media legislation. These domains were thoroughly examined to gain insights into participants’ attitudes, experiences, and perceptions related to news consumption and their interactions with digital media.

Themes were constructed based on a combination of codes and interpretations that emerged upon an extensive review of literature and analysis of prior data.

#### 3.2.2 Analysis

Thematic Analysis (TA) was selected for its methodological clarity and accessibility, making it suitable for the researchers with varying levels of familiarity with qualitative methods [52]. According to Braun and Clarke, TA focuses on identifying patterns within the data that enhance the theoretical or conceptual understanding of a research problem, rather than an in-depth familiarity with individual cases [53].

The analysis was carried out following the six phases of Braun and Clarke's model [53].

1. *Data Recognition:* Familiarization with data to gain initial understanding and insight.
2. *Initial Code Generation:* Two team members (Y.B., F.Ç) independently coded portions of the transcripts using the codes developed in the first phase and then modified the transcripts to ensure code consistency.
3. *Theme Search:* Identifying broader patterns of meaning emerging from the initial codes.
4. *Review of Potential Themes:* Refining and examining themes to ensure that they accurately represent the data.
5. *Defining and Naming Themes:* Clarifying the essence of each theme and assigning appropriate names.
6. *Report Production:* Compiling the final analysis into a coherent and structured report.

**Fig. 1:**
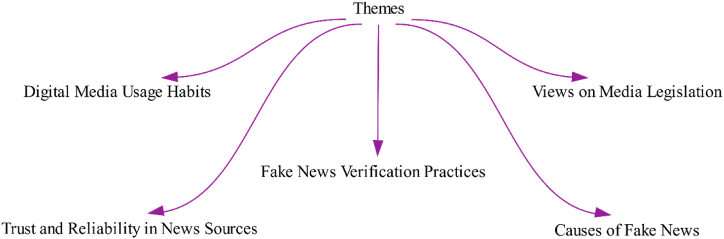
Themes.

In the study, we developed five main themes to broadly analyze attitudes, experiences, and perceptions related to digital media and news consumption, thus resulting in understanding the dynamics of societal media usage. As illustrated in Fig. 1, these themes offered a structured framework for determining the multifaceted relationship between society and digital media and highlighting the diverse ways in which people engaged with and perceived news in the digital age.

Theme 1 Digital Media Usage Habits: This theme explored the frequency of digital media use among participants, their preferred platforms, and their news-following habits.

Theme 2 Trust and Reliability in News Sources: This theme examined the degree of trust participants toward different news sources and the platforms they found reliable.

Theme 3 Fake News Verification Practices: This theme investigated deeply how individuals identified and verified fake news, particularly in the context of the earthquake

Theme 4 Causes of Fake News: This theme investigated the social and political impacts of fake news, including its psychological and behavioral effects on society.

Theme 5 Views on Media Legislation: This theme covered the participants' opinions on media laws and effectiveness of these laws.

#### 3.2.3 Ethical considerations

Ethical approval was obtained from the Akdeniz University, Social and Humanities Scientific Research and Publication Ethics Committee (Approval Number: 216, April 13, 2023). At the beginning of the study, the participants were informed about its purpose and scope, what they said would be used as data for the research, the academic and professional background of the research team as well as their motivations for conducting this study. They were also informed that they had the right to withdraw from the study at any time and the interviews would be recorded. Their permission was obtained to assure that all findings would remain confidential. All the subjects participating in in-depth interviews signed a written informed consent. This study adhered to the ethical guidelines recommended by the AAoIR Ethics Working Committee, specifically the ‘Ethical Decision-Making and Internet Research (Version 2.0) Recommendations.

## 4 Results

### 4.1 Theme 1: digital media usage habits

**Fig. 2:**
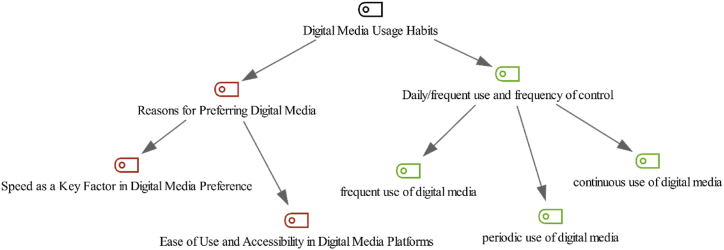
Digital media usage habits - code-subcode-sections model.

Th theme “Digital Media Usage Habits,” delved into the multifaceted aspects of how digital media platforms are utilized today, exploring the underlying motivations in such usage. Within this theme, we identified two primary sub-themes: “Reasons for Preferring Digital Media” and “Daily/Frequent Use and Frequency of Control”. Each sub-theme was further dissected into more specific subcodes to demonstrate different dimensions of digital media usage habits. These themes and subcodes are illustrated in Fig. 2.

**Fig. 3:**
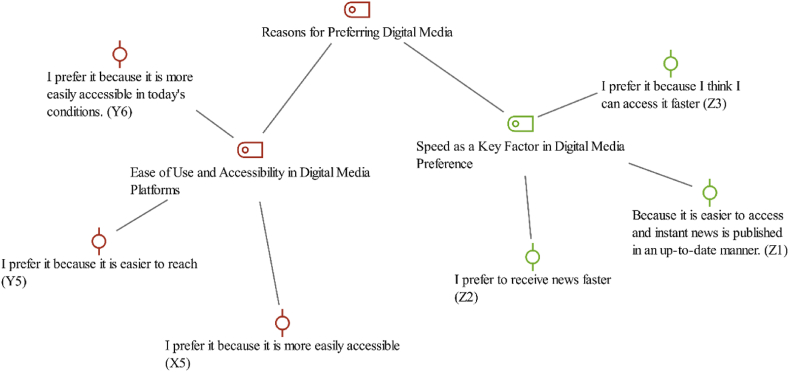
Reasons for preferring digital media- code-subcode-sections model.

The sub-theme “Reasons for Preferring Digital Media” delved into the motivations behind users’ preference for digital platforms, identifying “Ease of Use and Accessibility in Digital Media Platforms” and “Speed as a Key Factor in Digital Media Preference” as two pivotal factors, as illustrated in Fig. 3.

The participants’ comments highly illustrated these motivations, underscoring the importance of accessibility and speed in their preference for digital media. For instance, the participants Y1, Y5, and X5 emphasized the significance of accessibility by saying that

*“My preference is digital news sites because they are up-to-date and provide instant news.”* (Y1)

“I prefer it because it is easier to reach” (Y5).

In the context of digital media usage habits, the participants from Generation Y (Millennials) exhibited a clear preference for digital media, largely due to its up-to-date nature and speed.

These remarks highlighted how digital platforms' ease of access in the contemporary digital landscape is a crucial factor driving their preference. Similarly, X5's comment,

“I prefer it because it is more easily accessible” (X5)

reinforces the value attached to the easy accessibility of digital media.

Speed in accessing information was another critical aspect highlighted by the participants, particularly from Generation Z. Z2 stated:

*“I prefer to receive news faster”* (Z2)

Z1 observed that digital media was preferred.

“Because it is easier to access and instant news is published in an up-to-date manner” (Z1), and “I prefer it because I think I can access it faster” (Z3)

All of the statements highlighted the pivotal role of speed of access to information. These comments reflected a broader trend among digital media users, especially younger ones, who prefer to obtain information fast and stay abreast of the latest news through digital platforms.

Conversely, the sub-theme “Daily/Frequent Use and Frequency of Control” analyzed how digital media is integrated into daily life by examining the frequency and manner in which these platforms were utilized. This analysis uncovered a number of user engagement, categorized under subcodes of “Periodic Use of Digital Media,” “Continuous Use of Digital Media,” and “Frequent Use of Digital Media.” These classifications indicated the diverse patterns of digital media interaction, ranging from intermittent checking to nearly uninterrupted online presence, across different generational cohorts.

The present study revealed significant intergenerational differences in digital media usage habits. The participants from Generation Z (Gen Z), such as Z4, often described maintaining a nearly constant online presence. For instance, the Z4's remark,

“I’m always online, browsing apps and staying updated through Facebook, news channels with notifications on.” (Z4),

highlighted their consistent reliance on digital platforms for accessing news content. This statement exemplified a contemporary approach to staying informed, emphasizing the convenience and immediacy of digital news consumption that is prevalent among Generation Zindividuals.Moreover, the participant's utilization of diverse applications and notifications for news updates signifies a notable transition toward digital media within the modern information landscape.

Similarly, the Participant Z5 said:

*“I frequently browse social media, although not exclusively for news,”* (Z5),

and thus emphasized their regular yet multifaceted engagement with social platforms as well as how younger generations, particularly Generation Z, seamlessly integrate social media into their daily routines for various purposes along with just news consumption. It underscored a comprehensive digital lifestyle where news consumption represents only one dimension of their broader social media involvement, indicating a seamless combination of information gathering and social interaction in their digital practices.

The participants from Generation X exhibited an elaborate and intentional approach to their digital media consumption. For instance, the Participant X2 said,

*“I usually check my favorite news sites in the morning and evening. I like being informed but don’t need to be constantly connected.”* (X2),

by indicating a preference for a structured approach to access news. Another participant from the same generation, X4, deepened this understanding:

*“I check frequently during the day, but for traditional news sites versus digital ones, it’s only once or twice a day. Unfortunately, our phones, the drug of our age, are always at hand, allowing fast access to everything”* (X4).

These insights draw a portrait of Generation X as adeptly balancing traditional news habits with the efficiencies of the digital era by demonstrating a selective and purpose-driven use of digital media.

Comments of the participants illustrated their trust in the reliability of digital news sources. These opinions from the participants in Generation Y emphasized a pronounced shift toward digital platforms for news, driven by desires of speed, accessibility, and accuracy. This generational shift highlighted the evolving media consumption in the digital age.

These generational differences in digital media use shed light on the changing nature of media interaction. Generation Z's almost innate integration with digital platforms suggests a high level of comfort and dependence on technology, possibly as a result of growing up in a digitally rich environment. On the other hand, Gen Xers' engagement was more deliberate and structured, reflecting their adaptation to digital platforms at a later stage in their lives. Millennials oscillated between these two approaches and exhibited a multifaceted but selective engagement with digital media.

### 4.2 Theme 2: trust and reliability in news sources

**Fig. 4:**
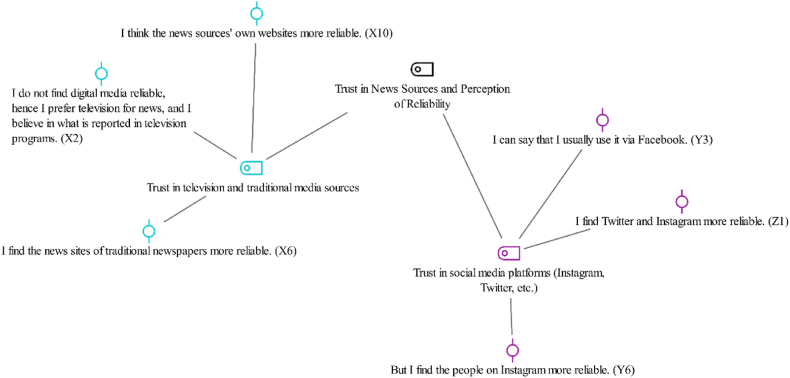
Trust and reliability in news sources-code-subcode-sections model.

The present study addressed this theme under two sub-themes: trust in television and broadcast media sources, and trust in social media platforms such as Instagram and Twitter (Fig. 4).

Findings of the present study about participants’ trust in news sources and their perceptions of reliability highlighted varied attitudes across generations.

Gen Xers had a clear preference for using broadcast media sources. Many exhibited trust in television and traditional news platforms by emphasizing reliance on more conventional methods of news consumption. The participant X2 expressed this view by stating,

“*I do not find digital media reliable, so I prefer television for news and believe what is reported there*.” (X2).

This view was repeated by the Participant X6, who noted,

“*I find the news sites of traditional newspapers more reliable.”(*X6)

These statements reflected a blend of trust in broadcast media and reliance on personal networks for news verification among Gen Xers.

The Millennial Generation (Generation Y) generally found news from digital platforms to be reliable, although some exhibited a selective trust. For instance, the participants Y6 and Y3 said;

“*But I find the people on Instagram more reliable.”*(Y6),

*“I find it generally reliable. I follow the pages of news sites on Facebook,”* (Y3).

Their statements indicated trust in specific digital platforms rather than a blanket trust across all.

Similarly, the trust in news sources among the participants from Generation Z reflected the trust among those from Generation Y. The participant Z1 emphasized their trust in Twitter and Instagram, stating,

*“I find Twitter and Instagram more reliable.”* (Z1)

This indicated that Generation Z had a tendency to trust social media platforms by valuing their immediacy and direct user interaction. Likewise, the Participant Z10 expressed a similar view by stating,

*“I don’t find them all reliable. I trust people on Instagram more,”* (Z10)

This view illustrated Generation Z's tendency to place greater trust in individual curators or influencers on platforms like Instagram, perhaps due to perceived authenticity or relatability.

These opinions suggested that Generation Z did not equally trust all digital news sources. Instead, they exhibited a selective trust, preferring platforms that combined the rapid dissemination of news with a sense of community and personal connection.

The participants from Generation X had a clear preference for broadcast media sources and trust in television and traditional news platforms. This reliance on more conventional methods of news consumption suggested a blend of trust in broadcast media and personal networks for news verification. On the other hand, Millennial (Gen Y) and Generation Z found digital news sources trustworthy but exhibited a selective trust. Overall, findings of the present study highlighted the importance of understanding generational differences in news consumption habits and the need for tailored approaches in addressing information dissemination and trust-building strategies across different age groups.

4.3. Theme 3: Fake News Verification Practices.

**Fig. 5:**
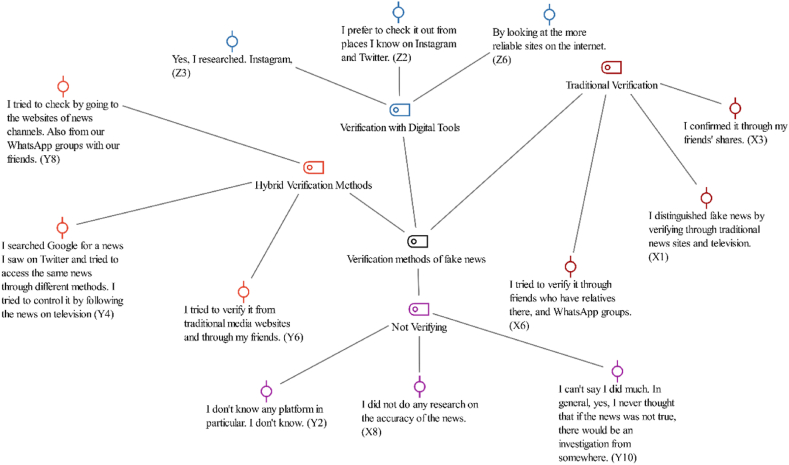
Fake news verification practices- code-subcode-sections model.

Theme 3 “Fake News Verification Practices,” examined the methods employed by individuals to verify news content by categorizing these approaches into four sub-codes: “Hybrid Verification Methods,” “Verification with Digital Tools,” “Traditional Verification,” and “Not Verifying.” Fig. 5 shows these sub-codes.

The approaches of Gen Xers toward verifying fake news during the earthquake were predominantly compatible with traditional methods. Most of them relied on the opinions of their friends for verification with following comments,

“*I confirmed it through my friends' shares*” (X3), and

“*I tried to verify it through friends who have relatives there, and WhatsApp groups*” (X6).

Some participants utilized both traditional news sites and television to discern the authenticity of news encountered on social media. X1 stated,

“*I distinguished fake news by verifying through broadcast news sites and television.”* (X1),

“*I prefer to check through links on broadcast news sites.*” (X5).

Only one participant reported using a fact-checking website learned from one of his friends:

“*I used**Teyit.org**, a site recommended by a friend, a few times*” (X4).

These responses indicated the Generation X's tendency toward friends and WhatsApp groups for news verification, while also using traditional news sources and illustrating their reliance on conventional approaches for discerning truth in critical situations like earthquakes.

The Millennial respondents took various approaches to verify fake news, often by combining digital and traditional methods. The participant Y1, for instance, used fact-checking websites and official news channel web pages, highlighting reliance on digital verification tools:

“*I used**Teyit.org**and the official web pages of news channels for verification*” (Y1).

Similarly, the participant Y4 proactively sought multiple digital sources for verification:

“*I searched Google for news I saw on Twitter and tried to access it through different methods, including television”* (Y4).

Some participants like Y3 and Y7 combined digital checks with traditional methods, such as consulting friends and television:

“*I asked my friends for verification and checked it on television*” (Y3);

“*Some friends verified and I learned from them, too*” (Y7).

Some participants (Y5, Y8, and Y9) stated that they did not perform any verification because they trusted the platforms they followed or they did not know that they needed to verify. In contrast, Y10 and Y2 showed reliance on selected sources without further verification:

“*I didn’t verify much myself*” (Y10);

“*I didn’t verify any news*” (Y2).

Such responses from Millennials suggested a combined use of digital and traditional methods for news verification and demonstrated how they navigate in the complex information environment of the digital age.

The participants from Generation Z used a blend of personal networks and digital platforms in their approaches to verify fake news. The participant Z9, for example, relied on conversations with his friends:

*“I had friends in the earthquake zone, and I learned the situation from them”* (Z9).

Z6 preferred to trust online sites for fact-checking:

*“I checked fake news on websites I found more reliable”* (Z6).

The participant Z2 chose to use social media platforms for verification by stating,

*“I prefer to check on Instagram and Twitter among sources I know*” (Z2).

Finally, Z1 utilized both social media and search engines:

*“I checked fake news on Instagram, Twitter, and Google*” (Z1).

These responses illustrated the Generation Z's reliance on a combined use of personal networks and digital resources to navigate in the challenging environment of digital misinformation.

When examining in the context of the 2023 earthquake, the participants from different generations exhibited distinct approaches to discerning and validating digital information. Generation X tended to rely on broadcast media and personal networks for verification. The Millennials blended digital and traditional methods by utilizing fact-checking websites, social media, and their personal contacts. Generation Z leaned more toward digital platforms by combining personal networks with social media and online resources. These patterns reflected varying levels of digital literacy and trust in media sources, highlighting the need for enhanced media literacy in navigating the complex landscape of digital information.

### 4.3 Theme 4. causes of fake news

**Fig. 6:**
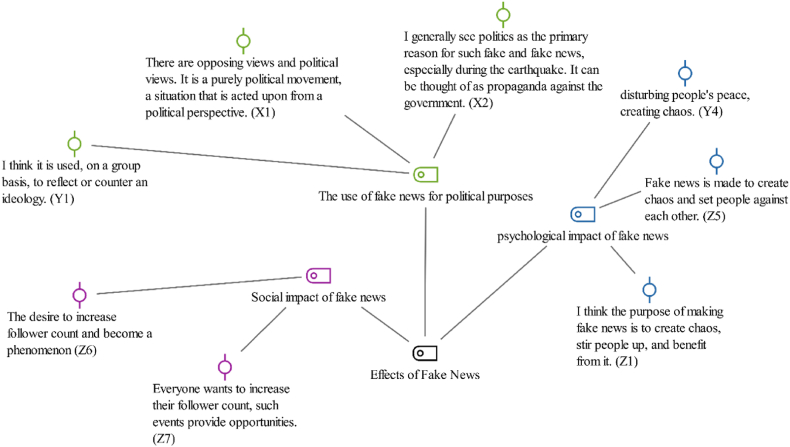
Causes of fake news- code-subcode-sections model.

Theme 4 “Causes of Fake News” was divided into three sub-themes: the utilization of fake news for political purposes, the psychological impact of fake news, and the social impact of fake news. Fig. 6 visually shows these sub-themes.

This theme examined how fake news affects society and its manipulation for specific ends. The sub-theme of utilization of fake news for political purposes examined how fake news is employed by political entities, particularly during significant events like elections. The sub-theme of psychological impact of fake news addressed the effect of these news items on interpersonal and community relations. Lastly, the interaction of influencers and social media indicated the dissemination and dynamics of fake news through popular figures and social media platforms, highlighting that fake news is not just misinformation but a tool for achieving specific social and political objectives.

The participants from the Generation X predominantly perceived fake news, especially in the context of natural disasters, as being driven by political motives. They stated that such misinformation was often used as a form of propaganda against the government or for political gain. The participant X1 said,

“*There are opposing political views; it is purely a political movement from a political perspective.”* (X1),

while X2 expressed,

“ *I see politics as the main reason for such fake news, especially during the earthquake; it can be seen as propaganda against the government”* (X2).

Another participant, reinforced this view by stating,

“*I believe that the spread of fake news is primarily for political outcomes*” (X10).

Additionally, some participants pointed to the manipulative aspect of misinformation, by suggesting its use in instilling fear and psychologically affecting people. For example, X9 said,

“*I think it’s to scare people and mess with their psychology*” (X9)

and X7 noted,

“*It’s about creating fear and psychologically affecting people*” (X7).

Overall, such responses of Generation X highlighted a common perception that fake news is a tool used for manipulating politically people and spreading fear among them.

The Millennial participants shared various views on the social and political impacts of fake news. Their views pointed to a strong belief in political motivations behind the spread of misinformation, especially in contexts of natural disasters or political events. For example, the Participant Y1 stated that he believed that misinformation is used strategically for ideological wars by stating,

“ *I think that fake news is used politically to project one's own ideology on the other side or to reject the opposing ideology.”* (Y1*)*

Some participants, such as Y7 and Y9, directly linked fake news to political agendas, saying,

“ *I think they used it to turn people against each other and serve for a political purpose.”* (Y7) and

“*I think it’s political*. *They expressed their opinion*” (Y9).

Regarding the timing, the Participant Y10 said,

“*It is used for political purposes, especially right before the elections*” (Y10).

Other participants expressed their concerns that fake news is being used to fuel social unrest or manipulate public opinion. One of the participants said,

“*It may be about provoking people more and creating chaos”* (Y2),

Y3 and Y4 shared their opinion by stating

“*It is disturbing people’s peace, creating chaos*” (Y3 and Y4).

This opinion pointed to a broader concern about the social impact of misinformation.

Y8 offered a different perspective, suggesting that the quest for popularity on social media could also increase the spread of fake news:

“*I think it’s more about people wanting to increase their follower count*” (Y8).

These responses from Millennials underscored a complex understanding of the role of fake news in modern society as well as the multifaceted impact of misinformation, perceiving it as a tool for political manipulation, social disruption, and personal gain in the digital age.

Generation Z's views on the social and political impacts of fake news highlighted a complex understanding. A great part of them emphasized the role of social media influencers and the pursuit of increased follower counts as primary motivators behind the spread of fake news. For example,

“*Everyone wants to increase their follower count, such events provide opportunities*” (Z7)

“*The desire to increase follower count and become a phenomenon*” (Z6).

This reflected a concern about the effect of social media popularity on the propagation of misinformation. Most of the participants focused on the role of misinformation in fostering chaos and social unrest. For example,

“*I think that the purpose of making fake news is to create chaos, stir people up, and benefit from it*” (Z1)

“*Fake news is produced to create chaos and set people against each other*” (Z5).

Only a minority (e.g., Z4) attributed these actions to political motives.

Generation Z's responses indicated their acute awareness of how fake news can be weaponized for personal gain, social disruption, and political agendas, highlighting the need for critical media literacy in the digital age.

The participants from Generation X primarily perceived fake news, particularly in the context of natural disasters, as a mechanism for political manipulation, suggesting that misinformation frequently serves as propaganda. This view revealed the strategic deployment of fake news for political objectives, highlighting its role in influencing public perception and governmental trust.

Conversely, the Millennials offered a more comprehensive analysis of fake news, acknowledging not only the political underpinnings but also its broader social repercussions. Their opinions revealed concern about how fake news can cause social unrest and manipulate public opinion, - specifically emphasizing its use to provoke and generate chaos, (the participants Y2, Y3, and Y4). They shed light on the multifaceted impact of fake news, extending beyond political manipulation to encompass significant social concerns.

Generation Z's contributions to the conversation further diversified the understanding of fake news by highlighting the effect of social media influencers and the quest for popularity as key drivers behind its proliferation. This generation also recognized the capacity of fake news to exacerbate social unrest by underscoring its role in creating discord and division within communities.

Moreover, these generational views enriched the discourse on fake news, illustrating a shift from viewing it solely as a tool of political manipulation to recognizing its extensive implications for society at large. The insights emphasized the complex dynamics at play in the dissemination and reception of fake news.

### 4.4 Theme 5: views on media legislation

**Fig. 7:**
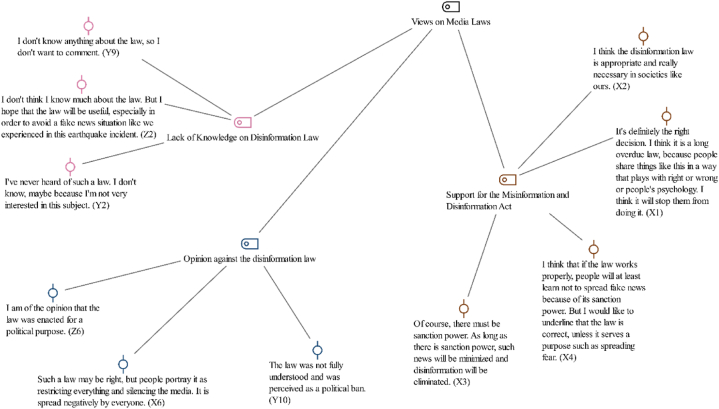
Views on media legislation- code-subcode-sections model.

In the modern digital landscape, the proliferation of disinformation has prompted legislative bodies worldwide to enact laws aimed at taking this phenomenon under control. Theme 5 delved into the public's attitudes toward the Disinformation Law, by considering various opinions that ranged from staunch opposition to strong support. This theme was divided into three primary subcodes: “Opinion against the Disinformation Law,” highlighting the concerns and criticisms from those who believe that such laws may infringe on free speech and civil liberties; “Support for the Misinformation and Disinformation Act,” capturing the viewpoints of individuals advocating for legal measures as necessary tools to combat misinformation; and “Lack of Knowledge on Disinformation Law,” pointing to a significant gap in public understanding and awareness of the specifics and implications of these laws (Fig. 7).

In the context of media legislation, Generation X's views showed a significant support for the disinformation law, but there were small differences in their views. The participant X1 appreciated the law's timing and necessity by stating

“ It’s definitely the right decision. I think it is a law deemed as necessary for a long time, because people share things like this in a way that plays with right or wrong or people’s psychology. I think it will stop them from doing such posts” (X1).

Another participant, X2, repeated this view by emphasizing the necessity of the law in modern societies:

‘I think that the disinformation law is appropriate and really necessary in societies like ours … ’ (X2).

Moreover, X3 put emphasis on the importance of sanctions in minimizing misinformation, suggesting,

“ Of course, there must be sanction power. As long as there is sanction power, such news will be minimized and disinformation will be eliminated “(X3).

These comments reflected a consensus among Generation X that the law is a vital step toward regulating digital content, with a focus on the need for effective implementation and enforcement to ensure its success in combating misinformation.

The Millennials (Generation Y) exhibited diverse opinions on the disinformation law. Y1 supported the law by countering claims of infringement on freedoms:

“I find this law’s enactment correct, particularly as I disagree with the notion that it restricts freedoms of thought and expression.” (Y1).

The participant Y5 mentioned about the law as follows:

“It is a completely political law and I think it is not necessary.” (Y5).

Y4, on the other hand, stated that he was skeptical about the effectiveness of the law:

“If the law was effective, there would be no such fake posts” (Y4).

Y10 highlighted misunderstandings about the law's purpose by indicating that it was perceived as a political censorship.

“The law was not fully understood and was perceived as a political ban.” (Y10).

These different views among millennials indicated a nuanced understanding and acceptance of disinformation law, differing based on their interpretations and concerns about the consequences and effectiveness of this law.

Generation Z exhibited a largely positive stance toward the disinformation law, with a notable majority endorsing its importance. For example, Z7 viewed it as crucial for preventing the spread of false news.

“It’s a necessary law, at least that’s how false news doesn’t happen” (Z7).

Similarly, Z8 appreciated its role in safeguarding people's mental well-being during delicate periods by regulating news content.

“Good in terms of preventing news made without considering people’s psychology” (Z8).

On the other hand, there were opposite views, as exemplified by Z9, who perceived the law as a potential restriction on freedom, along with concerns about its interpretation and application.

“Everyone interprets the law differently … more as a restriction” (Z9).

The examination of attitudes toward the disinformation law across Generation X, Millennials (Generation Y), and Generation Z revealed a complex tapestry of opinions that underscored the multifaceted nature of this legislation's reception. The participants from Generation X generally viewed the law as a necessary measure to regulate digital content. Millennials, however, displayed various attitudes, from acknowledging the law's significance in protecting freedoms of thought and expression to critiquing it as unnecessary or politically motivated. This diversity reflected a detailed understanding among Millennials, shaped by concerns over the law's consequences and its perceived effectiveness in combating fake news. Generation Z's largely positive stance toward the law indicated a broader endorsement of its objectives. Yet, dissenting voices within Generation Z highlighted apprehensions about the law's potential to impinge on freedom and the need for careful interpretation and application.

This comparative analysis across generations indicated the dynamic interplay between the perceived necessity of the disinformation law and concerns about its implications for freedom and effectiveness. It put an emphasis on the critical challenge of providing the balance between regulatory measures and the preservation of democratic values in the digital age, as well as the importance of a detailed legislation that addresses misinformation while safeguarding individual liberties.

The findings of the present study are summarized in Table 2 to provide a comprehensive overview of the differences and similarities among Generations X, Y, and Z.

**Table 2:** Summary of the findings by thematic analysis and generation.

Gen	Theme 1 Media Use/Habit	Theme 2 Trust and Reliability	Theme 3 Fake News Verification Practices	Theme 4 Causes of Fake News	Theme 5 Views on Media Legislation
**X**	Selective use; prefers structured news intake; balances broadcast and digital media (X2, X4)	Strong preference for broadcast media; television and traditional news platforms are trusted (X2, X6)	Relies on traditional methods and personal networks for verification; uses traditional news sites and television (X1, X3, X4, X5, X6)	Perceives fake news as politically motivated, used for propaganda, and psychologically manipulative (X1, X2, X10, X9, X7)	Strong support for disinformation law; sees it as necessary for regulating digital content and combating misinformation (X1, X2, X3)
**Y**	High preference for digital media due to its up-to-date nature and speed; accessibility is crucial (Y1, Y6, Y5)	Trusts digital platforms selectively; prefers specific platforms like Instagram and Facebook for news (Y6, Y3)	Blends digital and traditional methods; uses fact-checking websites, social media, and personal contacts for verification (Y1, Y3, Y4, Y7, Y10, Y2)	Views fake news as politically driven, used for social unrest, manipulation, and gaining social media popularity (Y1, Y6, Y7, Y9, Y10, Y2, Y3, Y4, Y8)	Diverse views; support and skepticism regarding disinformation law; concerns about its effectiveness and political motivations (Y1, Y5, Y4, Y10)
**Z**	Nearly constant online presence; relies heavily on social media; values speed and accessibility (Z2, Z1, Z3, Z4, Z5)	Selective trust in social media platforms; values immediacy and user interaction on platforms like Twitter and Instagram (Z1, Z10)	Combines personal networks and digital platforms for verification; uses social media, search engines, and trusted online sites (Z1, Z2, Z6, Z9)	Highlights the role of social media influencers and popularity in spreading fake news; recognizes its use in creating chaos and societal unrest (Z7, Z6, Z1, Z5, Z4)	Generally positive stance toward disinformation law; sees it as crucial for preventing false news and protecting mental well-being, with some concerns about restrictions (Z7, Z8, Z9)

## 5 Discussion

This study focused on the February 6, 2023 Earthquake in Türkiye and how this disaster was perceived through the media as well as examining generational differences and digital media usage habits. The findings of the present study indicated significant differences among generations in terms of digital media usage habits. These differences helped us better understand how media consumption habits and attitudes toward news are shaped, particularly during large-scale and impactful events such as the Türkiye's February 6, 2023 earthquake. In particular, the constant online presence of Generation Z and their ways of integrating digital media into their daily lives were effective in their responses to earthquake news and fake news. Likewise, the studies by Panagiotou et al. [54], and Mude and Undale [55] also reported how the media consumption habits of this generation have evolved. As stated by the participants Z4 and Z5, the conventional relationship of Generation Z with digital media reflected their raising in a technology-based environment. Their constant online presence and diverse application usage are compatible with the literature portraying them as ‘digital natives’ who seamlessly integrate digital platforms into their lives, as discussed by Prensky [19] and Palfrey & Gasser [56].

On the other hand, in the study by Gitnux [57] it was reported that Generation X's more selective and goal-oriented media consumption evaluated news with a balanced approach. This approach, observed in participants X2 and X4, was characterized by selective media consumption, especially for news updates. This means that this generation, having experienced the technological transition process during their adult years, displayed a more critical and measured attitude toward earthquake news [57]. Generation Y exhibited a combination of both behaviors, valuing reliable information while turning to digital platforms. The participant Y1's preference for digital media due to its immediacy and Y3's trust in its accuracy exemplified the Millennial generation's tendency toward digital platforms while valuing reliable information. This reflected the portrayal of millennials as a transitional generation, adept at navigating both digital and broadcast media environments, which is also emphasized in studies by Euajarusphan [58].

The present study shed light on the social and political effects of fake news in large-scale crises such as the Turkey's February 6, 2023 Earthquake. The participants from the Generation X in particular perceived fake news as a tool of political manipulation. As noted in Egelhofer and Lecheler's [59] study, the use of misinformation by political entities to manipulate public opinion became more evident during the spread of earthquake news. This reflected broader social trends where news consumption is often influenced by political beliefs.

As emphasized in the study by Stroud [60], individuals’ tendency to prefer news sources in line with their political leanings may affect their access to information and verification practices in times of crisis such as earthquakes [60]. This is consistent with the statements of Generation X participants that ideological congruence can affect trust in news sources.

Furthermore, in their study, Van Aelst et al. [61], suggested that news trust can be influenced by political leanings. This supported the participant X1's view that news produced during the earthquake had an ideological motive. On the other hand, studies on media bias have revealed the complexity of ideological tendencies in news consumption. For example, Stone [62] examined how consumers and journalists ideologically interpreted information and its effects on news consumption [62]. This suggested that the participants from Generation X may evaluate news based on their own ideological biases. The millennials thought that fake news was used as a tool to create social unrest and manipulate public opinion during the earthquake. The use of fake news for social disruption and public opinion manipulation, especially during crises, is supported by findings of the study by Starbird et al. [63], indicating that fake news was used to manipulate public discourse.

On the other hand, the participants in Generation Z pointed out that fake news during the earthquake was used to increase social media popularity and followers. This finding supported the findings of the study by Marwick and Lewis [64]. In their study, Pérez-Escoda et al. [25], also highlighted the spread of fake news among young people and how it spreads rapidly through social media. As stated in their study, social media, which is considered the natural habitat of young people (especially Generation Z), provides a favorable environment for the spread of fake news. Generation Z's emphasis on personal gain and social disruption through fake news during the earthquake is compatible with the study by Lazer et al. [65], which highlighted the increasing sophistication of digital misinformation [65], and the studies by Vosoughi et al. [66], Tkhostov et al. [23], and Alfred and Wong [67], which revealed the role of social media in the rapid spread of misinformation.

The present study also mentioned about dangerous disinformation. During the February 6, 2023 earthquake in Türkiye, some participants stated that misinformation about the magnitude of the earthquake and the effectiveness of emergency response efforts were widely circulating on social media, particularly during crises or critical events that could cause significant public harm. In their study, Starbird et al. [63], reported that this misinformation led to confusion, fear, and distrust among the people [63]. The participants also highlighted various sources of political propaganda on Turkish social media. such as government officials, political parties, and foreign organizations that spread misinformation to influence public opinion or weaken political opponents. Vosoughi et al. [66], gave specific examples where propaganda was used to manipulate public perception during political events or crises in order to polarize society or distract from pressing issues [66].

The findings of the present study are compatible with broader social trends. As noted in the study by Althaus & Tewksbury [68], some Generation X participants stated that fake news were produced for spreading fear, especially during crises such as earthquakes. Such news is designed to capture viewers' attention and evoke their emotional responses. This approach shows the importance of emotional content in news consumption and its impact on audience perceptions. X6's statement “It is produced to spread the psychology of fear” is compatible with the findings of the study by Brants and Van Praag [69] reporting that emotional content in news can affect audience perceptions and trust [69].

Furthermore, in the study by van Prooijen [70] it was suggested that political misinformation can create social paranoia [70]. This illustrates how the media can become powerful tools during major events, such as earthquake, and their psychological and emotional impact on society. This finding suggested that fear and psychological impact play an important role in news consumption and can affect audience perceptions and trust. The present study also examined intergenerational differences in trust in news sources and news verification practices in the February 6, 2023 Earthquake in Türkiye. In particular, Generation Z participants tended to verify news through social media platforms and their friends. This reflected the growing effect of social networks on news consumption, especially among younger audiences, as noted by Hermida et al., [71]. Z1 and Z6's statements on verifying news using Instagram, Twitter, and trusted web pages highlighted the changing nature of news trust by suggesting that broadcast media sources have been augmented or replaced by social media networks as in the study by Metzger and Flanagin [24].

In the earthquake, Gen Xers placed more trust in broadcast media sources, while Millennials took a balanced approach, using both digital and broadcast media as a means of verification. Generation Z, on the other hand, showed more trust in specific digital platforms, especially social media. This suggested that trust in news sources evolved with the changing media environment and generational experiences, which was in line with Chadwick's generational news consumption theory.

The present study also examined generations’ responses to disinformation laws in the case of a large-scale crisis such as the February 6, 2023 earthquake in Türkiye. Generation X, which witnessed the transition from broadcast media to digital media, supported disinformation legislation. This may be because they see media legislation as a necessary tool to maintain order and credibility in the media environment.

Media legislation and legal frameworks are important to maintain standards of credibility and accuracy, especially in a rapidly changing digital media. Generation X's support for these regulations may reflect their concerns about the quality and reliability of media content. In addition, this generation has experienced first-hand the rapid changes in media technologies and their impact on society.

Millennials’ varying reactions to the law can be analyzed in light of their upbringing in the digital age. Their differing views may reflect a struggle between appreciating the immediacy and accessibility of digital media and accepting the potential for misinformation.

Generation Z, who has grown up in a fully digital environment, takes a critical approach to the concept of media legislation. Their perspectives may be shaped by their own experiences related to the rapid evolution of digital media and its impact on information dissemination. Their in-depth understanding of the complexities of digital media may influence their perceptions of such legislation.

This study focused on the perception of disinformation, the methods of countering it, and examining intergenerational differences in depth. The media consumption habits of different generations and their attitudes toward fake news, especially during a major crisis such as the February 6, 2023 earthquake in Türkiye, would make an important contribution to the related literature. Gen Z's methods of interacting with the media showed how the strategies used to combat disinformation differed compared to the Gen X and Millennials preferences of broadcast media consumption.

In addition to the concepts of multiple literacies highlighted by Valverde-Berrocoso et al. [5], and Wardle and Derakhshan [4], the present study revealed the critical role of media and information literacies, as well as news and data literacy, in combating disinformation. These findings revealed the challenges and opportunities that individuals face in accessing and verifying information, especially in the digital age, which is compatible with the finding of Southwell et al. [72], about need for dynamic media literacy education,

The present study offered new perspectives on the social and political effects of disinformation. The participants' views on the effects of disinformation revealed different perceptions and reactions across generations. This suggested that the impact of disinformation on society is limited to content but also closely related to individuals’ media consumption habits and critical thinking skills.

The importance of media legislation has become even more apparent during major events such as earthquakes. The digital age requires reassessment and updating of media laws and regulations, while the transition from broadcast media environments to digital platforms has brought new challenges and opportunities. This transformation requires media laws and regulations to be adapted to reflect the characteristics and needs of the digital media environment. An effective media legislation must balance the protection of information integrity with the protection of freedom of expression and meet the changing needs of a digitally diverse society.

In conclusion, this study focused on different generations’ views on media legislation and anti-disinformation approaches as well as the power of the media in times of crisis such as earthquakes and the social impact of fake news. It emphasized the importance of different approaches across generations in media consumption and combating fake news as well as the need for further in-depth studies on this issue.

### 5.1 Theoretical implications

The present study contributes to the theoretical understanding about intergenerational differences in media consumption and information verification in several ways. Firstly, it highlights the small differences between different generations interact and digital media during crises, deepening existing theories on media engagement. By examining how Generations X, Y, and Z use digital platforms to access and verify information, this study provides a detailed perspective on the evolving nature of media consumption habits. Secondly, it underscores the importance of considering generational context in misinformation and disinformation, suggesting that age-related differences play a crucial role in how information is processed and trusted. This expands the theoretical framework of media studies by integrating generational perspectives, which have often been overlooked in previous studies. Additionally, the study contributes to understanding the impact of sociopolitical context on media consumption and misinformation. By exploring how political pressures and potential self-censorship influence the behaviors and perceptions of different generations, this study adds a layer of complexity to existing media theories.

### 5.2 Practical implications

The findings of this study have several practical implications for media practitioners, policymakers, and educators. For media practitioners, understanding the different media consumption habits of Generations X, Y, and Z can give information about the development of targeted communication strategies during crises, ensuring that accurate information reaches all age groups effectively. Tailored messaging can enhance the credibility and trustworthiness of news dissemination. For policymakers, the study emphasizes the need for updated media laws and regulations that address the challenges of the digital age, particularly concerning misinformation and disinformation. Policies that balance freedom of expression with the need to prevent the spread of false information are crucial in today's media landscape. Finally, for educators, the study highlights the importance of media literacy education tailored to different generational needs. By fostering critical thinking skills and improving the ability to discern credible information from falsehoods, education can play a vital role in building information resilience among the public. Furthermore, this study provides valuable insights into how different generations perceive and interact with information during crises and how to develop strategies that address the specific needs and behaviors of each age group. In conclusion, this study offers both theoretical and practical contributions to the understanding of media consumption and misinformation across generations, emphasizing the need for deliberate, context-wise approaches to media engagement and literacy.

### 5.3 Limitations and future research

An important limitation of this study is that all participants had at least an associate's degree. Therefore, the findings may be largely limited to individuals with university and higher education levels. Furthermore, the qualitative methodology used in this study and the limited sample size limit the generalizability of the results. Despite the researchers' efforts to establish a diverse sample group, the specific nature of the methodology does not guarantee a representation of the entire Turkish population.

Additionally, there are potential political constraints and considerations that could influence both the researchers and participants. While academic freedom is highly valued, recent laws aimed at combating disinformation in Türkiye have raised concerns about their potential use to suppress legitimate criticism. This socio-political environment might have led some participants to exercise self-censorship, affecting the honesty of their responses.

Some participants expressed concerns about the misuse of censorship by the government.

The research team was mindful of these dynamics and took appropriate precautions to balance the integrity of the study with the prevailing socio-political environment. The participants were informed of their rights and assured of confidentiality to encourage open and honest responses. Despite these measures, it is important to note that some participants’ answers may have been influenced by self-censorship, reflecting a general cautious approach to sensitive issues.

Moreover, it is important to address the criticisms of the “Digital Native” concept, as proposed by Prensky (2001). Brown and Czerniewicz, (2010) stated in their study that while the term suggested that younger generations, particularly Generation Z, were inherently proficient with digital technologies, it faced significant criticism for oversimplifying the complexities of digital access and skills (Brown & Czerniewicz, 2010). In the context of Türkiye, not all individuals within Generation Z have equal access to digital media, especially among less affluent groups. The disparity in digital access, often referred to as “digital apartheid,” highlights the socio-economic barriers that prevent many young people from fully engaging with digital media. This study's focus on university-educated individuals signified that the participants likely represented more privileged segments of their generations, potentially overlooking the experiences of those with lower socio-economic backgrounds. Additionally, while the present study provides insights into the digital media usage of an educated elite, it does not account for the experiences of less affluent individuals who may not have the same opportunities in accessing to digital technologies. This limitation is critical, as it highlights the need for future studies to explore digital media usage across a broader socio-economic spectrum. Addressing this gap would provide a more comprehensive understanding of digital media consumption and misinformation across different segments of the population.

However, the compliance of the results with similar international studies suggests that the identified issues and problems are not exclusive to our context. Therefore, we believe that our findings contribute significantly to the international literature.

The qualitative nature of this study offers rich and detailed insights, allowing us to understand the complex social dynamics of the participants’ experiences and perceptions, as explained by Tracy (2019) [73].

Future studies could benefit from similar qualitative approaches to further explore these dynamics in different regions and among individuals with different educational and socio-economic backgrounds. This approach could enhance the generalizability of the findings and provide a better understanding of intergenerational differences in media consumption habits and attitudes toward fake news. Additionally, complementing qualitative research with quantitative studies on similar topics could validate the findings on a larger scale. Further studies exploring the relationship between media consumption habits and digital literacy levels in depth would be beneficial. Such studies could provide deeper insights into intergenerational differences and information about the development of effective policies and practices in media consumption and combating fake news. Furthermore, local studies can be conducted to better understand differences within a generation (whether class, cultural, ethnic, religious) and across them. These local studies can build on our current research and contribute to a more comprehensive understanding of these dynamics.

## Funding

The authors did not receive any funding for this study.

## Ethical approval

Ethical approval was obtained from Akdeniz University, Social and Humanities Scientific Research and Publication Ethics Committee (Approval Number: 216, April 13, 2023).

## Informed consent

All participants in this study gave their written consent by signing the informed consent form.

## Data availability statement

The dataset analyzed in this study is not publicly available due to the subjects’ permission to use it only for data analysis and not for any other use, but it is available from the corresponding author upon a reasonable request.

## Additional information

Correspondence and requests for materials should be addressed to Fatma Çakmak.

## CRediT authorship contribution statement

**Yasemin Bilişli:** Writing – review & editing, Writing – original draft, Validation, Supervision, Project administration, Methodology, Investigation, Formal analysis, Data curation, Conceptualization. **Fatma Çakmak:** Writing – original draft, Validation, Methodology, Investigation, Formal analysis, Data curation, Conceptualization. **Selin Aygen Zetter:** Investigation, Data curation. **Mehmet Ilgaz Ünal:** Investigation, Data curation.

## Declaration of competing interest

The authors declare that they have no known competing financial interests or personal relationships that could have appeared to influence the work reported in this paper.
