# Supplementary material for: Navigating truth and disinformation: A comparative analysis of generational responses to the 6 February 2023 earthquake in digital media in Türkiye
Source: Heliyon. 2024 Sep 27;10(19):e38667. doi: 10.1016/j.heliyon.2024.e38667 (PMC11471220; doi:10.1016/j.heliyon.2024.e38667)
Supplement: Multimedia component 1 [file mmc1.docx]

| **Topic** | **Questions** |
| --- | --- |
| Digital Media Usage and News Consumption | Do you use digital media to get news?  How often do you use it?  Why do you prefer digital news sites?  Do you find digital news sites reliable?  Which channels do you trust? |
| Encounters and Reactions to Disinformation | Did you encounter any fake/false news about the earthquake?  How did you identify them as fake?  What platforms and topics were involved in the fake news you encountered?  Did you verify the accuracy of the news you got about the earthquake?  Which platforms do you use for verification? |
| News Verification Practices | How do you verify the authenticity of a suspicious image/photo/video?  Have you ever used news verification platforms? Which ones did you use? |
| Causes and Effects of Fake News | What do you think are the reasons for producing fake/false news during the earthquake?  Would you share a digital news item without verifying it?  Please share your views on the effects of fake/false news and the disinformation law enacted to combat them. |

**Supplementary materials**

**Table S1.** The interview guide
