# Supplementary material for: Navigating truth and disinformation: A comparative analysis of generational responses to the 6 February 2023 earthquake in digital media in Türkiye
Source: Heliyon. 2024 Sep 27;10(19):e38667. doi: 10.1016/j.heliyon.2024.e38667 (PMC11471220; doi:10.1016/j.heliyon.2024.e38667)
Supplement: Multimedia component 2 [file mmc2.docx]

**Table S2.** The COREQ form

| **Topic** | **Item no** | **Guide Questions/Description** | **Reported on**  **page no** |
| --- | --- | --- | --- |
|  | 1. Interviewer/facilitator | Which author/s conducted the interview or focus group? | 9 |
|  | 2. Credentials | What were the researcher’s credentials? | 9 |
|  | 3. Occupation | What was their occupation at the time of the study? | 9 |
|  | 4. Gender | Was the researcher male or female? | 1 |
|  | 5. Experience and training | What experience or training did the researcher  have? | 9 |
| Domain 1: Research  team and reflexivity |  |  |  |
|  | 6. Relationship established | Was a relationship established prior to study  commencement? | 8 |
|  | 7. Participant knowledge of the interviewer | What did the participants know about the  researcher? e.g. personal goals, reasons for doing the research | 10 |
|  | 8. Interviewer characteristics | What characteristics were reported about the interviewer facilitator? e.g. Bias, assumptions, reasons  and interests in the research topic | 9 |
|  | 9. Methodological orientation and Theory | What methodological orientation was stated to  underpin the study? e.g. grounded theory, discourse analysis, ethnography, phenomenology, content analysis | 6 |
|  | 10. Sampling | How were participants selected? e.g. purposive,  convenience, consecutive, snowball | 7 |
|  | 11. Method of approach | How were participants approached? e.g. face-to-  face, telephone, mail, email | 8 |
|  | 12. Sample size | How many participants were in the study? | 7 |
|  | 13 Non-participation | How many people refused to participate or dropped  out? Reasons? | 8 |
|  | 14. Setting of data collection | Where was the data collected? e.g. home, clinic,  workplace | 8 |
| Domain 2: Study design | 15. Presence of non-  participants | Was anyone else present besides the participants  and researchers? | 8 |
|  | 16. Description of sample | What are the important characteristics of the  sample? e.g. demographic data, date | 7 |
|  | 17. Interview guide | Were questions, prompts, guides provided by the  authors? Was it pilot tested? | 6 |
|  | 18. Repeat interviews | Were repeat inter views carried out? If yes, how  many? | 7 |
|  | 19. Audio/visual recording | Did the research use audio or visual recording to  collect the data? | 10 |
|  | 20. Field notes | Were field notes made during and/or after the  interview or focus group? | 8 |
|  | 21. Duration | What was the duration of the inter views or focus  group? | 8 |
|  | 22. Data saturation | Was data saturation discussed? | 8 |
|  | 23. Transcripts returned | Were transcripts returned to participants for  comment and/or corrrection | 8 |
|  | 24. Number of coders | How many data coders coded the data? | 9 |

|  | 25. Description of the coding  tree | Did authors provide a description of the coding  tree? | 9 |
| --- | --- | --- | --- |
|  | 26. Derivation of themes | Were themes identified in advance or derived from  the data? | 9 |
|  | 27. Software | Derivation of themes | 6 |
|  | 28. Participant checking | Did participants provide feedback on the findings? | 8 |
| Domain 3: analysis  and findings | 29. Quotations presented | Were participant quotations presented to illustrate  the themes/findings? | 11-21 |
|  | 30. Data and findings  consistent | Was there consistency between the data presented  and the findings? | 11-21 |
|  | 31. Clarity of major themes | Were major themes clearly presented in the  findings? | 11-21 |
|  | 32. Clarity of minor themes | Is there a description of diverse cases or discussion  of minor themes? | 11-21 |
